# Advances in Neuroprosthetic Learning and Control

**DOI:** 10.1371/journal.pbio.1001561

**Published:** 2013-05-21

**Authors:** Jose M. Carmena

**Affiliations:** Department of Electrical Engineering and Computer Sciences, Helen Wills Neuroscience Institute, University of California, Berkeley, California, United States of America

## Abstract

Significant progress has occurred in the field of brain–machine interfaces (BMI) since the first demonstrations with rodents, monkeys, and humans controlling different prosthetic devices directly with neural activity. This technology holds great potential to aid large numbers of people with neurological disorders. However, despite this initial enthusiasm and the plethora of available robotic technologies, existing neural interfaces cannot as yet master the control of prosthetic, paralyzed, or otherwise disabled limbs. Here I briefly discuss recent advances from our laboratory into the neural basis of BMIs that should lead to better prosthetic control and clinically viable solutions, as well as new insights into the neurobiology of action.

## Abstract 2

This essay summarizes recent advances in the field of brain-machine interfaces, with a focus on the learning and acquisition of neuroprosthetic skills.

## Introduction

The goal of cortically controlled motor neuroprosthetics [1]–[14] is to reliably, accurately, and robustly convey enough motor control intent from the central nervous system (CNS) to drive multi–degree-of-freedom (DOF) prosthetic devices by patients with amputated, paralyzed, or otherwise immobilized limbs for long periods of time (decades). To achieve this goal, two main challenges remain: 1) how to make viable neural interfaces that last a lifetime, and 2) skillful control and dexterity of a multi-DOF prosthetic device comparable to natural movements. In a BMI system, neural signals recorded from the brain are fed into a machine that transforms these signals into a motor plan. This is the subject's “intention of movement,” which is then streamed to the prosthetic device. A closed control loop is established by providing the subject with visual and sensory feedback of the prosthetic device.

The first challenge is to have a neural interface viable for a lifetime. In the front end, the physical substrate should be able to withstand a variety of biotic and abiotic effects that presumably lead to performance degradation at the electrode-tissue interface [15]. In the back end, the system should be wireless, require minimum power, and support bidirectional dataflow, i.e., “reading” and “writing” from/to the brain. Ideally, these systems would be fully implantable in the intracranial space as well as have batteryless operation. They should also be modular enough to allow the measurement and stimulation of different types of neural signals, such as the electrical activity of individual neurons or groups of neurons, as well as other physiological parameters such as glucose, brain pulsation, etc. that may become important for powering the implanted device in future generations of this technology [16].

The second challenge is getting the brain to recognize an “actuator,” or prosthetic device that is not part of the body, and being able to control it without enacting overt physical movements (as in the case of a paralyzed patient). This has two differentiated components: motor and sensory. On the sensory side, the goal is to provide realistic sensory feedback from the prosthetic device by directly stimulating sensory areas in brain regions that would mimic lost/damaged inputs. This should allow the user to *feel* the environment through the prosthetic device, which has been supported by recent examples using electrical microstimulation [17],[18]. Future BMI systems may incorporate optical stimulation in lieu of electrical stimulation [19]. On the motor side, we suggest that in order to boost the performance of current BMI systems both neural adaptation (brain plasticity) and artificial adaptation (machine learning) should be combined in a coadaptive way. Ultimately, the goal is to achieve a quantum-leap increase in neural controllable degrees of freedom that should allow a patient to effortlessly perform tasks of daily living.

Next we will focus on recent advances, mostly from our laboratory, that are relevant to the acquisition and retention of skills to control disembodied effectors such as computer cursors and prosthetic limbs. These advances gravitate around the concept of “prosthetic motor memory” facilitated through brain plasticity, and the “tuning” of decoding algorithms while the subject is using the BMI.

## Decoding Natural Actions vs. Learning to Perform New Ones

When thinking about BMI design, there are at least two different approaches one could take for converting thought into action, also known as the decoding vs. learning argument [20],[21]. One approach aims to decode (or read out) the natural motor plan to control the missing, impaired, or intact limb. In this approach, a mathematical model or decoder that relates neural activity to natural limb movements is generated and then used to predict these movements from the recorded neural activity alone. The other approach requires the brain to learn a transform in order to control the new actuator, irrespectively of physical movement of the natural limb. This approach treats a BMI system as a “modified CNS” that has to be learned.

But why should we approach BMI as a modified CNS? When interfacing the brain with a machine, we are effectively creating a de novo circuit for action. The neuroprosthetic system under control in this new circuit is fundamentally different than the natural system used to control the native arm. For instance, our musculoskeletal systems have very little to do with robotic limbs in the way they function and how they are controlled. The same applies to the spinal cord, which in the neuroprosthetic system is approximated by a set of mathematical rules called the transform. This transform projects from a high dimensional space of dozens to hundreds of neurons to a subspace of a few control signals (e.g., position and velocity of the end effector). This is particularly important because the motor and sensory pathways will be compromised in patients with spinal cord injury or other neurological disorders. Hence, if we are trying to control a prosthetic device that is different from our native arm, why should we aim to *decode* the brain signals related to this arm in the first place? Instead, could the brain *learn* to control a prosthetic device that is not part of the natural body and generate novel actions with it?

One important aspect relevant to this discussion is the type of experimental model used for BMI experiments. The brunt of the invasive BMI work (i.e., that which uses implantable technology to record from populations of neurons) is currently done in able-bodied animal subjects [22] except for some exceptions in animal models of spinal cord injury [23], temporary models of paralysis induced via nerve blocks [9],[11],[12], as well as a few clinical trials in humans [7],[13],[14]. In addition, there is an increasing number of studies in epileptic and stroke patients that involve BMI tasks using electrocorticographic (ECoG) signals [24],[25]. So, how can able-bodied subjects learn these circuits for neuroprosthetic control? Our approach is to change the rules of the instrumental learning task previously learned under manual control.

Take for example a standard center-out reaching task in which the subject manually controls an actuator—a robotic manipulandum, exoskeleton, or its own natural limb—to reach for instructed targets in order to obtain a reward. The performance feedback received by the subject typically consists of a visual observation from a computer screen displaying the controlled actuator. Upon switching to neural control (or “BMI mode”), the experimenter swaps the visual feedback from the actuator controlled manually with that of the actuator controlled through the BMI. Here is when the manner in which the subject is instructed to perform the BMI task is key. By physically removing the actuator from the experimental rig during BMI mode (or restraining the arm to the primate chair in the case of the natural arm being the actual actuator used to reach for targets), we are effectively changing the rules of the task. It is no longer a “move joystick to center target” type of rule but one that requires learning to mentally steer the actuator using biofeedback. This triggers a learning process that we call “transform learning” [26], in which the brain modifies the tuning properties of the neurons incorporated into the BMI (and therefore causally linked to behavior) to minimize error in the motor output through a process of plasticity [10],[27],[28].

Alternatively, not changing the rules of the task upon switching to BMI mode (that is, keeping the experimental setup and task the same as during manual control) typically leads to the able-bodied subject continuing to engage as in the manual task (i.e., overtly moving the natural limb and oblivious that a change of mode of operation, from manual to BMI mode, has taken place). Thus, as the afferent and efferent pathways in the subject remain intact, the patterns of neural activity evoked during BMI mode will be very similar to those evoked during manual control. This mode of BMI operation predicts few plastic changes in the brain, since the same circuits for motor control are being used.

## Circuit Stability Facilitates Prosthetic Motor Memory

As noted, in the learning approach to BMI control, the subject has to learn the “spinal cord” (i.e., the transform) for neuroprosthetic function in order to perform the actions required to achieve the desired goals. For practical reasons, this learning process is typically initialized with a biomimetic transform (i.e., generated from natural arm movement data). However, this is not a requirement, as previous work has shown that primates and even rodents can also learn arbitrary transforms trained with nonbiomimetic data [9],[10],[28],[29], demonstrating the capacity of the brain to create de novo circuits to perform novel (neuroprosthetic) actions.

Regardless of the way in which the transform is trained (biomimetic or not), the complexity of both the prosthetic device to be controlled (e.g., degrees-of-freedom of the apparatus) and the task to be performed play a crucial role in transform learning. For instance, in early studies [2]–[4], new transforms were trained at the beginning of every session. In this approach, the subject has to effectively learn a new transform every day before being able to perform the task proficiently. If the task is simple enough, the brain can learn the transform in a single day (intrasession). However, as task complexity increases, it becomes more difficult for the subjects to learn the daily trained transforms. This results in variable performance from day to day that prevents consolidation and retention of prosthetic skill [10],[26]. Hence, intrasession transform learning alone becomes impractical for learning skillful neuroprosthetic control. So, how can we achieve consolidation of the learned skill?

We hypothesized that pairing stable neural recordings with a fixed, static transform—as opposed to retraining the transform every day—would lead to retention of the learned skill across time and therefore facilitate the consolidation of a prosthetic motor memory. The key element here is the stability of the circuit; the neural input to the transform and the parameters of the transform remains unchanged throughout learning. This is what we tested in previous work [10],[27] in which we showed that the primate brain can achieve and consolidate skilled control of a prosthetic device in a way that resembles that of natural motor learning, i.e., a motor skill that is retained. Specifically, when a fixed transform algorithm was applied to stable recordings from an ensemble of primary motor cortex (M1) neurons across days, there was dramatic long-term consolidation of prosthetic motor skill. This process created a motor map for prosthetic function that was readily recalled and remarkably stable across days. Surprisingly, the same set of neurons could learn and consolidate a second motor map without interference with the first map, highlighting another attribute of transform learning that is similar to natural motor learning: that of being able to learn new motor skills without interfering with previously acquired skills.

Hence, transform learning leads to the formation of a stable cortical map that has the putative attributes of a memory trace; namely, it is stable across time, readily recalled, and resistant to interference. We believe such a *prosthetic motor memory* will be critical for the skillful control of multi-DOF prosthetic limbs, and that these devices could eventually be controlled through the nominally effortless recall of motor memory in a manner that mimics natural skill acquisition and motor control.

## The Role of Machine Learning in the BMI Loop

**Figure 1:**
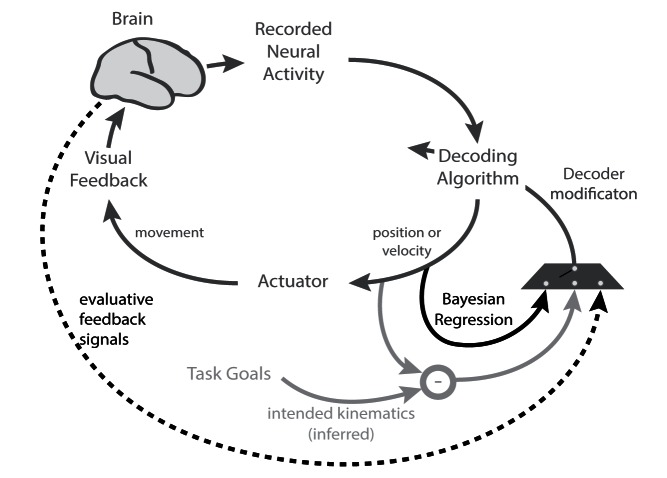
Closed-loop decoder adaptation (CLDA) accelerates learning and improves performance by updating a BMI decoder's parameters in closed-loop operation (i.e., while the subject is using the BMI). The gray arrows point to the main elements of a closed-loop BMI: sensing (neural activity), estimation (decoding algorithm or transform), control of the actuator, and feedback. The red arrows represent the CLDA component. BMI errors are analyzed online with respect to inferred or known task goals, and/or on evaluative feedback. These errors are used to modify the decoder's parameters. Overall, CLDA improves BMI performance by making the decoder more accurately represent the true underlying mapping between the user's neural activity and their intended movements (adapted from [30] with permission).

What about adaptation taking place in the machine instead of the brain? This process, known as closed-loop decoder adaptation (CLDA) [30], is an emerging paradigm for achieving rapid performance improvements in BMI control (Figure 1). CLDA consists of adapting the transform's or decoder's parameters during closed-loop BMI operation (i.e., while the subject is using the BMI) to more accurately represent the mapping between the user's neural activity and their intended movements [3],[31]–[36]. The error signals required to adapt the decoder can be estimated in a variety of different ways, including using the task goals to infer the subject's intention [33], Bayesian methods to self-train the decoder [34], and extracting error signals directly from the brain [35].

The design process of a CLDA algorithm requires important decisions not only about *which* parameters of the decoder should be adapted and *how* these should be adapted, but also *when*, (i.e., how often), as the rate at which the decoder changes can influence performance. Also important is the way in which the decoder is initialized. Movement disorders such as paralysis and stroke prevent patients from making the types of natural movements that are often used to initiate the decoder. As a result, less favorable methods of decoder initialization, such as motor imagery, must be used, typically resulting in low initial performance. To address the problem of accelerating learning and boosting BMI performance in these settings, we recently developed SmoothBatch, a CLDA algorithm that improves performance in a relatively short time and independent of the decoder initialization conditions [36]. This method infers the subject's intended movement goals during online control [33] and updates the decoder on an intermediate (1–2 min) time-scale. The main feature of SmoothBatch is that it can readily improve performance in a relatively short time, independent of the subject's initial closed-loop BMI performance. This could be particularly useful in clinical applications in which the patient cannot move the limbs.

While CLDA algorithms can readily improve performance in a relatively short time, the brain still faces a “moving-target” problem of being able to learn an adaptive decoder. Can we facilitate the coadaptation between the brain and the machine so that the motor memory–like properties emerging through *transform learning* can be preserved while adapting the transform? One possible avenue for future studies could be starting with an early CLDA phase, in which the transform is adapted until certain level of performance is achieved, followed by a prolonged period of static transform, allowing the brain to optimize its control.

## Conclusion

Achieving skillful control of a multi-DOF prosthetic will entail synergizing two different types of adaptation processes: natural (brain plasticity) and artificial (machine learning). In addition, providing realistic sensory feedback from the prosthetic device should allow the user to *feel* the environment and achieve more natural control. Transform learning facilitates the formation and retention of a prosthetic motor memory through a process of neuroplasticity. CLDA techniques expedite the learning process by adapting the transform during online performance. We believe that BMI systems capable of exploiting both neuroplasticity and CLDA will be able to boost learning, generalize well to novel movements and environments, and ultimately achieve a level of control and dexterity comparable to that of natural arm movements.

## Abbreviations

BMI: brain–machine interfaces
CLDA: closed-loop decoder adaptation
CNS: central nervous system
DOF: degree-of-freedom
ECoG: electrocorticography

## References

[1] Chapin JK, Markowitz R, Moxon KA, Nicolelis MAL (1999) Direct real-time control of a robot arm using signals derived from neuronal population recordings in motor cortex. Nat Neurosci 2: 664–670. 10404201 10.1038/10223

[2] Serruya MD, Hatsopoulos NG, Paninski L, Fellows MR, Donoghue JP (2002) Instant neural control of a movement signal. Nature 416: 141–142. 11894084 10.1038/416141a

[3] Taylor DM, Tillery SI, Schwartz AB (2002) Direct cortical control of 3D neuroprosthetic devices. Science 296: 1829–1832. 12052948 10.1126/science.1070291

[4] Carmena JM, Lebedev MA, Crist R, O'Doherty JE, Santucci DM, et al (2003) Learning to control a brain–machine interface for reaching and grasping by primates. PLoS Biol 1: e42 doi:10.1371/journal.pbio.0000042. 14624244 10.1371/journal.pbio.0000042 PMC261882

[5] Musallam S, Corneil BD, Greger B, Scherberger H, Andersen RA (2004) Cognitive control signals for neural prosthetics. Science 305: 258–262. 15247483 10.1126/science.1097938

[6] Santhanam G, Ryu SI, Yu BM, Afshar A, Shenoy KV (2006) A high-performance brain– computer interface. Nature 442: 195–198. 16838020 10.1038/nature04968

[7] Hochberg L, Serruya MD, Friehs GM, Mukand J, Saleh M, et al (2006) Neuronal ensemble control of prosthetic devices by a human with tetraplegia. Nature 442: 164–171. 16838014 10.1038/nature04970

[8] Velliste M, Perel S, Spalding MC, Whitford A, Schwartz AB (2008) Cortical control of a prosthetic arm for self-feeding. Nature 453: 1098–1101. 18509337 10.1038/nature06996

[9] Moritz CT, Perlmutter SI, Fetz EE (2008) Direct control of paralysed muscles by cortical neurons. Nature 456: 639–642. 18923392 10.1038/nature07418 PMC3159518

[10] Ganguly K, Carmena JM (2009) Emergence of a stable cortical map for neuroprosthetic control. PLoS Biol 7: e1000153 doi:10.1371/journal.pbio.1000153. 19621062 10.1371/journal.pbio.1000153 PMC2702684

[11] Suminski AJ, Tkach DC, Fagg AH, Hatsopoulos NG (2010) Incorporating feedback from multiple sensory modalities enhances brain–machine interface control. J Neurosci 30: 16777–16787. 21159949 10.1523/JNEUROSCI.3967-10.2010 PMC3046069

[12] Ethier C, Oby ER, Bauman MJ, Miller LE (2012) Restoration of grasp following paralysis through brain-controlled stimulation of muscles. Nature 485: 368–371. 22522928 10.1038/nature10987 PMC3358575

[13] Hochberg LR, Bacher D, Jarosiewicz B, Masse NY, Simeral JD, et al (2012) Reach and grasp by people with tetraplegia using a neurally controlled robotic arm. Nature 485: 372–375. 22596161 10.1038/nature11076 PMC3640850

[14] Collinger JL, Wodlinger B, Downey JE, Wang W, Tyler-Kabara EC, et al (2012) High-performance neuroprosthetic control by an individual with tetraplegia. Lancet 381: 557–564 doi:10.1016/S0140-6736(12)61816-9. 23253623 10.1016/S0140-6736(12)61816-9 PMC3641862

[15] Prasad A, Xue Q-S, Sankar V, Nishida T, Shaw G, et al (2012) Comprehensive characterization and failure modes of tungsten microwire arrays in chronic neural implants. J Neural Eng 9: 056015. 23010756 10.1088/1741-2560/9/5/056015

[16] Rapoport BI, Kedzierski JT, Sarpeshkar R (2012) A glucose fuel cell for implantable brain–machine interfaces. PLoS ONE 7: e38436 doi:10.1371/journal.pone.0038436. 22719888 10.1371/journal.pone.0038436 PMC3373597

[17] Venkatraman S, Carmena JM (2011) Active sensing of target location encoded by cortical microstimulation. IEEE Trans Neural Syst Rehabil Eng 19: 317–324. 21382769 10.1109/TNSRE.2011.2117441

[18] O'Doherty JE, Lebedev MA, PJ, Zhuang KZ, Shokur S, et al (2011) Active tactile exploration using a brain–machine–brain interface. Nature 479: 228–231. 21976021 10.1038/nature10489 PMC3236080

[19] Boyden ES, Zhang F, Bamberg E, Nagel G, Deisseroth K (2005) Millisecond-timescale, genetically targeted optical control of neural activity. Nat Neurosci 8: 1263–1268 doi:10.1038/nn1525. 16116447 10.1038/nn1525

[20] Jackson A, Fetz EE (2011) Interfacing with the computational brain. IEEE Trans Neural Syst Rehabil Eng 19: 534–541. 21659037 10.1109/TNSRE.2011.2158586 PMC3372096

[21] Fetz EE (2007) Volitional control of neural activity: implications for brain–computer interfaces. J Physiol 579: 571–579. 17234689 10.1113/jphysiol.2006.127142 PMC2151376

[22] Nuyujukian P, Fan JM, Gilja V, Kalanithi PS, Chestek CA, et al (2011) Monkey models for brain-machine interfaces: the need for maintaining diversity. Conf Proc IEEE Eng Med Biol Soc 2011: 1301–1305. 22254555 10.1109/IEMBS.2011.6090306

[23] Knudsen EB, Moxon KA, Sturgis EB, Shumsky JS (2011) Skilled hindlimb reaching task in rats as a platform for a brain-machine interface to restore motor function after complete spinal cord injury. Conf Proc IEEE Eng Med Biol Soc 2011: 6315–6318. 22255782 10.1109/IEMBS.2011.6091558

[24] Leuthardt EC, Schalk G, Wolpaw JR, Ojemann JG, Moran DW (2004) A brain–computer interface using electrocorticographic signals in humans. J Neural Eng 1: 63–71. 15876624 10.1088/1741-2560/1/2/001

[25] Bundy DT, Wronkiewicz M, Sharma M, Moran DW, Corbetta M, et al Using ipsilateral motor signals in the unaffected cerebral hemisphere as a signal platform for brain –computer interfaces in hemiplegic stroke survivors. J Neural Eng 9: 036011. 22614631 10.1088/1741-2560/9/3/036011 PMC3402181

[26] Ganguly K, Carmena JM (2010) Neural correlates of skill acquisition with a cortical brain–machine interface. J Mot Behav 42: 355–360. 21184353 10.1080/00222895.2010.526457

[27] Ganguly K, Dimitrov DF, Wallis JD, Carmena JM (2011) Reversible large-scale modification of cortical networks during neuroprosthetic control. Nat Neurosci 14: 662–667. 21499255 10.1038/nn.2797 PMC3389499

[28] Koralek KC, Jin X, Long JD, Costa RM, Carmena JM (2012) Corticostriatal plasticity is necessary for learning intentional neuroprosthetic skills. Nature 483: 331–335. 22388818 10.1038/nature10845 PMC3477868

[29] Fetz EE (1969) Operant conditioning of cortical unit activity. Science 163: 955–958. 4974291 10.1126/science.163.3870.955

[30] Dangi D, Orsborn AL, Moorman HG, Carmena JM (2013) Design and analysis of closed-loop decoder adaptation algorithms for brain-machine interfaces. Neural Comput In press. 10.1162/NECO_a_00460 23607558

[31] Gage GJ, Ludwig KA, Otto KJ, Ionides EL, Kipke DR (2005) Naive coadaptive cortical control. J Neural Eng 2: 52–63. 15928412 10.1088/1741-2560/2/2/006

[32] Shpigelman L, Lalazar H, Vaadia E (2008) Kernel-ARMA for hand tracking and brain-machine interfacing during 3D motor control. Adv Neural Inf Process Syst 21.

[33] Gilja V, Nuyujukian P, Chestek CA, Cunningham JP, Yu BM, et al (2012) A high-performance neural prosthesis enabled by control algorithm design. Nat Neurosci 15: 1752–1757. 23160043 10.1038/nn.3265 PMC3638087

[34] Li Z, O'Doherty JE, Lebedev MA, Nicolelis MA (2011) Adaptive decoding for brain-machine interfaces through Bayesian parameter updates. Neural Comput 23: 3162–3204. 21919788 10.1162/NECO_a_00207 PMC3335277

[35] Mahmoudi B, Sanchez JC (2011) A symbiotic brain-machine interface through value-based decision making. PLoS ONE 6: e14760 doi:10.1371/journal.pone.0014760. 21423797 10.1371/journal.pone.0014760 PMC3056711

[36] Orsborn AL, Dangi S, Moorman HG, Carmena JM (2012) Closed-loop decoder adaptation on intermediate time-scales facilitates rapid BMI performance improvements independent of decoder initialization conditions. IEEE Trans Neural Syst Rehabil Eng 20: 468–477. 22772374 10.1109/TNSRE.2012.2185066

